# Left-Sided Sciatic Nerve Endometriosis Presenting As Chronic Thigh Pain and Muscle Atrophy: A Case Report

**DOI:** 10.7759/cureus.108539

**Published:** 2026-05-09

**Authors:** Parviz Samadov, Kamil Aliyev, Ilhama Eldarova, Akbar Hajiyev, Emil Hasanov

**Affiliations:** 1 Radiology, Liv Bona Dea Hospital, Baku, AZE; 2 Gynecology, Liv Bona Dea Hospital, Baku, AZE; 3 Pathology, Liv Bona Dea Hospital, Baku, AZE

**Keywords:** catamenial sciatica, chronic thigh pain, deep infiltrating endometriosis, gemelli muscles, muscle atrophy, neuropathic pain, obturator internus muscle, pelvic mri, sciatic nerve endometriosis

## Abstract

Sciatic nerve endometriosis is a rare and often underdiagnosed form of deep infiltrating endometriosis involving the lumbosacral plexus or sciatic nerve. We present the case of a 40-year-old woman with chronic left thigh and pelvic pain exacerbated during menstruation, accompanied by limited mobility. Pelvic MRI revealed a lesion along the left sciatic nerve with characteristic hemorrhagic and inflammatory features, as well as muscle atrophy indicative of chronic denervation. Combined surgical and gynecologic intervention confirmed the diagnosis and resulted in significant symptom improvement. This case highlights the importance of clinical suspicion and MRI in early diagnosis to prevent irreversible neurologic damage.

## Introduction

Endometriosis is a prevalent gynecological disorder characterized by the presence of functional endometrial tissue outside the uterine cavity, primarily affecting pelvic organs, such as the ovaries, fallopian tubes, and peritoneum [1]. Although the condition commonly manifests with symptoms, including dysmenorrhea, chronic pelvic pain, and infertility, involvement of extrapelvic structures remains rare and diagnostically challenging [2]. Among these atypical presentations, sciatic nerve endometriosis represents a particularly uncommon and underrecognized form of deep infiltrating endometriosis, in which ectopic endometrial implants infiltrate or compress the lumbosacral plexus or sciatic nerve [3,4].

The clinical presentation of sciatic nerve endometriosis is often nonspecific and may mimic other neuropathic or musculoskeletal disorders, leading to frequent misdiagnosis and delayed treatment [5]. Patients typically report catamenial sciatica, characterized by cyclical neuropathic pain along the sciatic nerve distribution, accompanied by motor deficits and progressive muscle atrophy if left untreated [6,7]. The pathophysiological mechanisms underlying neural involvement are incompletely understood, and hypotheses include retrograde menstruation with peritoneal implantation, lymphovascular dissemination, direct extension from adjacent pelvic endometriotic lesions, and perineural invasion [8].

MRI has emerged as the imaging modality of choice for the detection of sciatic nerve endometriosis [9]. MRI findings typically include T1 hyperintense foci corresponding to blood products and T2 hypointense rims reflecting hemosiderin deposition or fibrosis, along with nerve thickening and surrounding inflammatory changes [10]. Early radiologic recognition is critical to guide timely surgical intervention and prevent irreversible neurologic impairment [11].

This report presents a rare case of left-sided sciatic nerve endometriosis, which is less common than right-sided involvement, given the predominance of right-sided involvement reported in the literature [9]. This case underscores the importance of maintaining a clinical suspicion in women presenting with catamenial sciatic pain and highlights the pivotal role of MRI in the diagnosis and treatment planning for optimal patient outcomes.

## Case presentation

We present the case of a 40-year-old woman with a two-year history of left thigh and pelvic pain, which had progressively worsened over the past seven months. The patient also experienced limited mobility and reported increased pain during menstruation. Previous physiotherapy interventions had exacerbated her symptoms. Lumbar disc pathology and nerve root involvement were considered, and an MRI of the pelvis was recommended.

MRI findings

Magnetic resonance imaging was performed on a Siemens 3.0-Tesla Magnetom Skyra system (Siemens Healthineers, Erlangen, Germany). The protocol included axial T1- and T2-weighted sequences; fat-suppressed T1-weighted and fat-suppressed T2-weighted sequences; and coronal and axial short-tau inversion recovery (STIR) imaging. Axial diffusion-weighted imaging (DWI) with corresponding apparent diffusion coefficient (ADC) maps was also obtained with coverage focused on the pelvic region.

**Figure 1:**
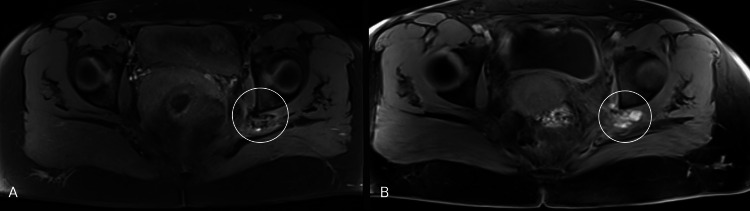
Left sciatic nerve lesion on T1 fat-suppressed MRI at six-month follow-up. (A) Initial T1 fat-suppressed image demonstrates a heterogeneous hyperintense lesion involving the left sciatic nerve, extending into the piriformis, obturator internus, and superior gemellus muscles (white circle). (B) Follow-up T1 fat-suppressed image at 6 months shows an increase in lesion size (white circle).

Pelvic MRI revealed a mass-like lesion along the left sciatic nerve at the level of the greater sciatic foramen. The lesion exhibited a T2 hypointense rim and central heterogeneous T1 hyperintense hemorrhagic components, extending into the piriformis, obturator internus, and superior gemellus muscles (Figures 1A-1B).

**Figure 2:**
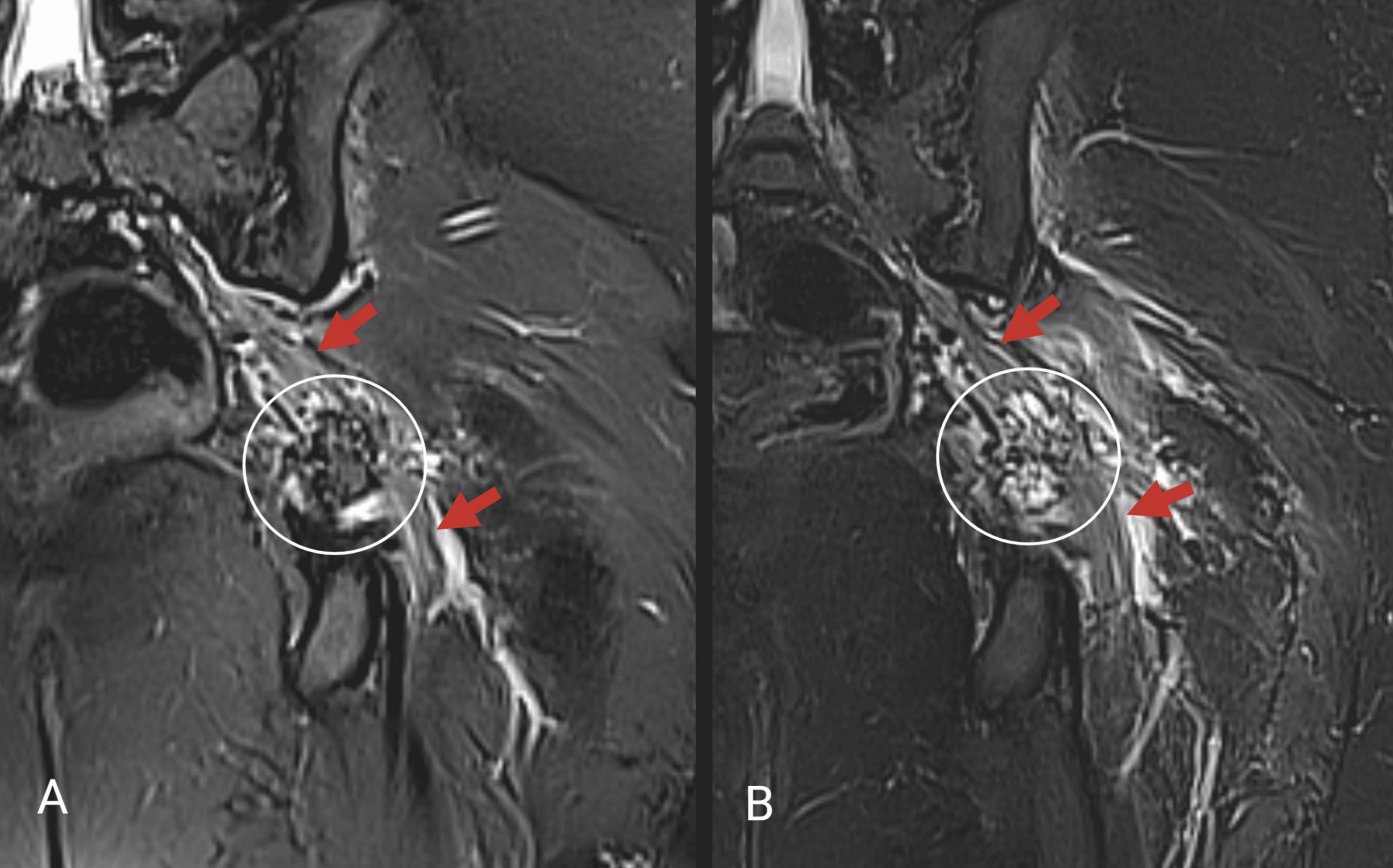
Coronal T2-weighted images of the left sciatic nerve lesion at six-month follow-up. (A) Initial coronal T2 image demonstrates the lesion (white circle), with a hypointense rim consistent with hemosiderin deposition. The sciatic nerve shows thickening along its course (red arrows). (B) Follow-up T2 image at six months shows lesion progression (white circle) and thickening along the sciatic nerve (red arrows).

The sciatic nerve was observed to be thickened and edematous, consistent with inflammation. MRI results further identified atrophy in the left gluteus maximus, superior and inferior gemellus, quadratus femoris, piriformis, and internal obturator muscles. Additionally, a T2/STIR hyperintense signal was noted, which may indicate denervation edema or inflammatory changes. Follow-up images after six months demonstrated disease progression and increasing muscle atrophy (Figures 2A-2B). 

**Figure 3:**
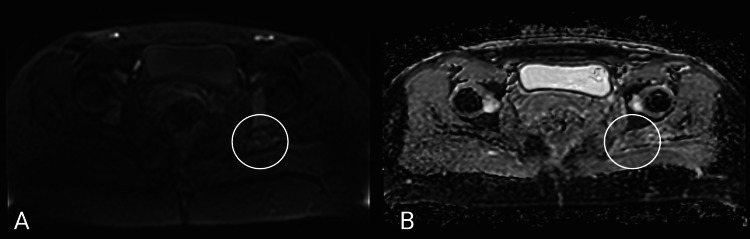
Diffusion imaging of the left sciatic nerve lesion. (A) Diffusion-weighted imaging (DWI) shows the lesion (white circle) without significant diffusion restriction. (B) The corresponding apparent diffusion coefficient (ADC) map confirms the absence of restricted diffusion.

Axial DWI and ADC map demonstrated no evidence of restricted diffusion within the lesion (Figures 3A-3B). The patient underwent combined surgical resection and gynecologic intervention.

**Figure 4:**
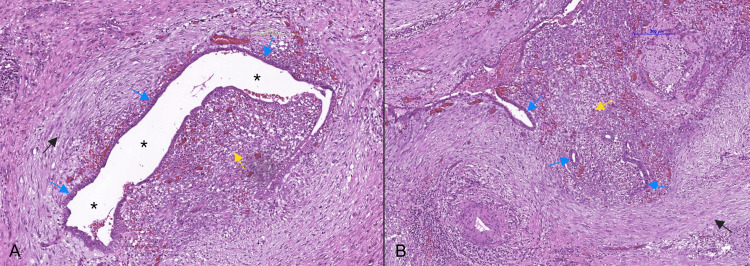
Histopathologic findings of endometriosis in deep soft tissue. (A) Low-power photomicrograph of H&E showing a dilated endometrial-type gland (black asterisk) lined by a single layer of cuboidal to columnar epithelium (blue arrows) and surrounded by compact endometrial-type stroma (yellow arrow) within the fibrocollagenous soft tissue (black arrow). (B) Low-power photomicrograph of H&E showing additional foci of endometrial-type glands (blue arrows) and stroma (yellow arrow) infiltrating fibroadipose tissue and skeletal muscle and extending around nerve bundles, accompanied by chronic inflammation, hemosiderin deposition, and fibrosis. No cytologic atypia or malignant features were observed. Scale bars = 200 μm.

Histopathological examination revealed fibroadipose and skeletal muscle tissue infiltrated by foci of endometriosis. The lesions were composed of endometrial-type glands and surrounding endometrial stroma embedded within the soft tissue, skeletal muscle fibers, and around nerve bundles. The glands were variably sized and lined by a single layer of cuboidal to columnar epithelium, without cytologic atypia. The surrounding endometrial-type stroma was composed of compact spindle cells resembling proliferative endometrial stroma. There was an associated chronic inflammatory infiltrate, scattered hemosiderin-laden macrophages, and areas of hemorrhage. Foci of fibrosis and reactive changes within adjacent skeletal muscle fibers were present, including mild fiber atrophy and separation by fibrous tissue. No cytologic atypia, complex glandular architecture, or features of malignancy were identified (Figures 4A-4B). Postoperative follow-up demonstrated significant symptom improvement and no residual lesions on MRI.

## Discussion

Sciatic nerve endometriosis represents a rare form of deep infiltrating endometriosis, characterized by the presence of ectopic endometrial tissue affecting the lumbosacral plexus or sciatic nerve. Although endometriosis predominantly affects pelvic organs, extrapelvic neural involvement is infrequent and often underdiagnosed due to nonspecific symptoms and delayed imaging assessments [1,2].

Patients typically exhibit catamenial sciatica, progressive neuropathic pain, and motor impairment. Consistent with previously documented cases, our patient experienced chronic pelvic and thigh pain, with exacerbation during menstruation, which serves as a critical clinical indicator for diagnosis [3,4]. Muscle atrophy and movement limitations have also been reported in cases with extended nerve involvement [5]. In our patient, atrophy of the obturator internus, gemelli, quadratus femoris, and gluteus maximus muscles, along with associated STIR hyperintensity, was indicative of chronic neurogenic denervation. Given the lesion's location at the greater sciatic foramen, these findings likely reflect involvement or compression of adjacent sacral plexus branches, including the nerve to obturator internus, the nerve to quadratus femoris, and the inferior gluteal nerve. Ipsilateral piriformis atrophy with STIR hyperintensity further supports denervation associated with deep infiltrating endometriosis at the sciatic notch region, including the nerve to piriformis (S1-S2).

Numerous studies indicate a predominance of right-sided sciatic nerve endometriosis [6], potentially attributable to pelvic anatomical structures and the patterns of peritoneal fluid circulation. One hypothesis suggests that the rectosigmoid colon, located in the left pelvis, may offer partial protection to left-sided extrapelvic structures, thereby reducing the likelihood of implantation on that side [9]. Contrarily, our patient exhibited left-sided sciatic involvement, a less frequently documented occurrence, thus contributing to the limited body of literature on left-sided cases.

A significant aspect of our case was the patient's history of physiotherapy, which exacerbated the pain. In the absence of gynecological correlation, sciatic endometriosis may initially be misdiagnosed as lumbar disc pathology or musculoskeletal disease, resulting in delayed diagnosis and inappropriate treatment [4,7]. In our patient, the catamenial pattern of pain and careful correlation with MRI signal characteristics facilitated early radiologic suspicion of endometriosis prior to surgical confirmation. MRI is currently the preferred imaging modality for assessing pelvic nerve involvement in endometriosis. Typical MRI features include T1 hyperintense foci due to blood products and T2 hypointense components corresponding to hemosiderin deposition or fibrotic tissue [3,8]. In our case, the lesion exhibited a T2 hypointense rim with a central heterogeneous signal and T1 hyperintense components, findings consistent with hemorrhagic content. Asymmetric thickening of the sciatic nerve and obliteration of the surrounding fat planes further supported neural infiltration. Serial imaging demonstrated lesion progression and increasing muscle atrophy, underscoring the importance of follow-up imaging in symptomatic patients.

The pathogenesis of sciatic nerve endometriosis remains a subject of debate. Proposed mechanisms include retrograde menstruation with peritoneal implantation, coelomic metaplasia, lymphovascular spread, and direct extension from adjacent pelvic endometriotic foci [5,11]. Additionally, neural spread along perineural planes has been described in magnetic resonance neurography studies [8,11]. Despite these theories, the precise mechanism underlying isolated neural involvement remains unclear.

Surgical excision is generally regarded as the primary treatment, particularly in cases presenting with progressive neurologic deficits [1,11]. Hormonal therapy, including gonadotropin-releasing hormone analogs, has demonstrated variable success, especially in perimenopausal women; however, it may not reverse established neural damage [7]. In our patient, a combination of surgical resection and gynecologic intervention resulted in significant postoperative pain reduction, with no evidence of residual lesions on follow-up MRI.

## Conclusions

Sciatic nerve endometriosis should be considered a potential diagnosis in women presenting with chronic thigh or pelvic pain, catamenial sciatica, and muscle atrophy. MRI is the preferred modality for early detection and facilitates timely surgical intervention. The occurrence of left-sided involvement is uncommon, highlighting the necessity for comprehensive imaging evaluation and clinical correlation to prevent misdiagnosis.
